# β-Catenin Knockout in the Olfactory Bulb and Hippocampus Reveals Dual Roles in Olfactory Discrimination and Working Memory

**DOI:** 10.3390/ijms27208945

**Published:** 2026-10-09

**Authors:** Yanzhi Liu, Mingpeng Kong, Hongkai Jin, Tsz Long Chu, Lee Wei Lim, Kathryn S. E. Cheah

**Affiliations:** 1 School of Biomedical Sciences, Li Ka Shing Faculty of Medicine, The University of Hong Kong, Hong Kong SAR, Chinakevinjhk715@hotmail.com (H.J.);; 2 Center of Excellence for Brain Health and Neurotechnology, Academy of Life and Natural Sciences, Xi’an Jiaotong-Liverpool University, Suzhou 215123, China; 3 Department of Neurosurgery, Maastricht University, 6229 HX Maastricht, The Netherlands; 4 Faculty of Medicine, Istanbul Atlas University, Istanbul 34408, Turkey

**Keywords:** β-catenin, olfactory bulb, odor discrimination, working memory, Wnt/β-catenin signaling

## Abstract

Mice rely heavily on their sense of smell to provide crucial environmental information. The intricate organization of neural circuits within the olfactory bulb (OB) and the communication across the OB-hippocampus network in mice underpin their ability to discriminate and adapt to odors and perform odor-guided behaviors. Wnt/β-catenin signaling has been shown to regulate neural stem cell proliferation and migration during embryogenesis and adult neurogenesis. However, the potential role of β-catenin in adult OB and hippocampus in olfaction, odor-guided behavior and working memory is not known. Here, we report the unexpected expression of collagen, type X, alpha 1 (*Col10a1*), in the postnatal mouse OB and the hippocampus starting at approximately postnatal day 14. We exploited this finding to generate OB and hippocampus conditional *Ctnnb1* knockout (*Ctnnb1*^cKO^) mice. We found *Ctnnb1*^cKO^ mice displayed impaired odor habituation and discrimination abilities, whereas food foraging ability was unaffected. Surprisingly, *Ctnnb1*^cKO^ mice displayed modestly improved working memory in the Radial Arm Maze test. Together, these findings identify behavioral phenotypes associated with *Col10a1*-Cre-driven *Ctnnb1* deletion and establish this mouse line as a useful tool for studying β-catenin function in adult OB–hippocampal circuits.

## 1. Introduction

Wnt/β-catenin signaling has indispensable roles in mammalian embryonic development [1], especially in osteogenesis [2], adipogenesis [3], and neurogenesis [4]. This pathway regulates neural plate development, neural stem cell proliferation and migration, axonal guidance, dendritic growth, and synapse formation [5,6]. Less is known about the roles of Wnt/β-catenin signaling in the adult brain. There is evidence that the canonical Wnt/β-catenin signaling regulates neural stem cell proliferation and fate commitment during adult neurogenesis [7,8,9,10,11]. In adult mammalian brains, neurogenesis persists throughout life in three discrete brain regions, olfactory neuroepithelium (OE), and subventricular zone (SVZ) and subgranular zone (SGZ) of the hippocampus [12]. The OE generates new olfactory receptor neurons (ORNs) that project axons to glomeruli in the bilateral olfactory bulbs (OB); the SVZ generates new progenitor neurons that migrate rostrally into the OB and differentiate into granule cells and periglomerular cells [12,13,14]; and the SGZ generates new neurons in the dentate gyrus (DG) of the hippocampus [15]. Notably, the two destinations of newborn neurons in the adult brain, the OB and the hippocampus, are integral parts of the neural network for olfaction and memory.

The OB-hippocampus axis is crucial for olfaction, olfaction-guided behaviors, and memory [16]. The OB is equipped with special neural circuits for the first order processing of olfactory signals. Odor molecules in nasal cavity are sensed by cilia of ORNs in the OE, generating neural inputs. Axons of ORNs expressing a specific type of odorant receptor undergo tremendous resorting and convergence after entering the OB and form synapses within glomeruli, where neural signals are transduced to mitral cells and tufted relay neurons [17]. Both cells further transduce the signals via axonal projections to the anterior olfactory nucleus, piriform (olfactory) cortex, amygdala, and entorhinal cortex [17,18,19]. The precise axonal convergence and spatial organization of glomeruli in the OB are essential for olfactory signal processing [20]. Habituation and dishabituation of odors depend on the adaption/recovery response of mitral cells in the OB [21]. Changes to the excitability of mitral/tufted neurons have been shown to affect odor discrimination in mice, as well as alter the whole-body metabolic profile [22]. Although acetylcholine release has been shown to dishabituate OB odor responses [23], the signaling pathways/genes regulating habituation/dishabituation of odors in the OB remain largely unknown. Odor discrimination requires the formation of neural representations and associations of specific odor inputs in the hippocampus [24,25]. In mice, the OB projects directly to the entorhinal cortex, which relays information to the hippocampus [17,18]. The hippocampus in turn provides top-down feedback to the OB via monosynaptic or polysynaptic connections [18,26,27]. The OB-hippocampus network shows coherent beta oscillations during odor discrimination [28]. The hippocampus serves as a central hub that coordinates communications from cortical and subcortical areas to form episodic memories that integrate sensory experiences, emotions, and contextual information [29].

However, there is limited knowledge of the molecular pathways involved in the establishment of bidirectional communication between the OB and hippocampus that underlies olfaction-guided behavior and memory. Since Wnt/β-catenin signaling regulates adult neurogenesis, which takes place in confined regions in the OB-hippocampus axis, it is plausible that Wnt/β-catenin signaling regulates OB-hippocampus dependent behaviors. During embryogenesis, Wnt/β-catenin signaling is essential for ORNs in the OE to form axonal contacts with the developing forebrain [30]. Wnt-responsive neural stem cells in the OB have been shown to differentiate into granule cells, calretinin-expressing periglomerular cells, and tyrosine hydroxylase-expressing periglomerular cells postnatally [31]. In adult mice, *Ctnnb1* is expressed in the OB and hippocampus at P56 [32], but little is known about the role of Wnt signaling in adult olfaction and memory mediated by the OB and hippocampus.

Collagen, type X, alpha 1 (*Col10a1*), encoding collagen X, is well known to be expressed in hypertrophic chondrocytes in developing endochondral bone [33,34]. In the present study, we discovered the unexpected expression of *Col10a1* in the OB and hippocampus of postnatal mice. We exploited the expression property of *Col10a1*-Cre to mediate ablation of *Ctnnb1* in the OB and hippocampus. Given the established roles of Wnt/β-catenin signaling in neural development and adult neurogenesis, we hypothesized that loss of β-catenin in OB–hippocampal circuits would alter olfactory-guided behavior and working-memory performance. We, therefore, characterized *Col10a1*-Cre reporter activity and assessed olfactory-guided behavior and spatial working memory in *Col10a1*-Cre-*Ctnnb1* conditional knockout mice (*Ctnnb1*^cKO^).

## 2. Results

### 2.1. Col10a1-Cre-Driven tdTomato Reporter Expression in the Olfactory Bulb and Hippocampus

**Figure 1:**
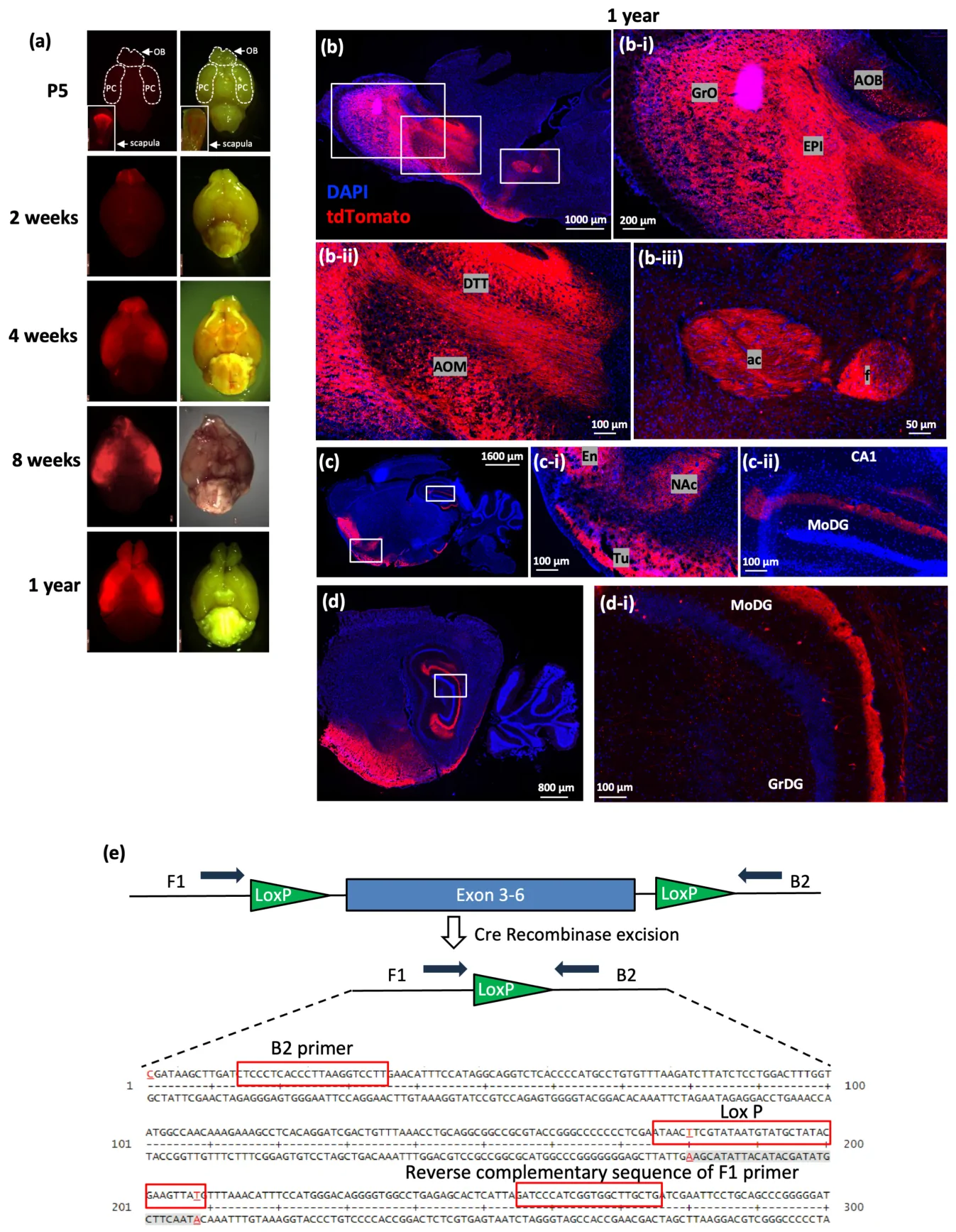
(**a**) *Col10a1-*Cre-driven tdTomato expression in *Col10a1^Cre/+^;R26^td/+^* reporter mice. TdTomato activity can be detected in olfactory bulb and piriform cortex during adolescent and adult stages. At P5, tdTomato activity was not detected in the brain; the expression of tdTomato in hypertrophic chondrocytes and their descendants in scapula is shown as a positive control of tdTomato activity [34] (lower left inset). (**b**,**d**) *Col10a1-*Cre-driven tdTomato expression (red) counterstained with DAPI (blue) in *Col10a1^Cre/+^; R26^td/+^*; *Ctnnb1^−/−^* mice at 1 year shown in sagittal sections. (**b**) tdTomato activity can be seen in the olfactory-related regions including the granular cell layer (GrO) and external piriform layer (EPI) of the MOB, the AOB, and extending to the dorsal tenia tecta (DTT), medial part of the anterior olfactory nucleus (AOM), anterior commissure (ac) and fornix (f). (**c**,**d**) TdTomato activity can be seen in surrounding brain regions including the endopiriform nucleus (En), nucleus accumbens (NAc), olfactory tubercle (Tu), and the molecular and granule cell layers of the dentate gyrus (MoDG and GrDG). Scale bars are as shown on the images. (**e**) Confirmation of Cre activity in excising exon 3-6 of *Ctnnb1* in *Ctnnb1* cKO mice. Upper panel: schematic of loxP flanked exons 3-6. *Col10a1*-driven Cre recombinase excises floxed exon 3-6 of *Ctnnb1* gene, leaving only one LoxP site in the genome which was verified by DNA sequencing (lower panel). The structure of *Ctnnb1-flox* allele was previously described [2]. Scale bars: (**b**), 1000 μm; (**b-i**), 200 μm; (**b-ii**), 100 μm; (**b-iii**), 50 μm; (**c**), 1600 μm; (**c-i**,**c-ii**), 100 μm; (**d**), 800 μm; (**d-i**), 100 μm.

To identify possible genes that could be exploited for genetic manipulation in mice to study adult olfaction and memory, we searched the in situ hybridization (ISH) database of the Allen Brain Atlas (https://mouse.brain-map.org/static/atlas, accessed on 1 July 2023) for genes expressed in the OB and hippocampus. We found that *Col10a1* mRNA was detected in the adult mouse brain in discrete regions including the mitral cell layer of the OB, the granule cell layer of the DG, and the pyramidal cell layer of the hippocampus [32]. We employed the *Col10a1^Cre/+^*; *R26^td/+^* reporter line [34,35] to determine the spatial-temporal expression pattern of *Col10a1* in postnatal mouse brain. At postnatal day 5 (P5), no obvious tdTomato reporter activity was observed. Beginning from postnatal day 14 (P14, 2 weeks), some tdTomato activity was observed in the OB. At 4 weeks, tdTomato signal was more intense in the OB than at P14. The piriform cortex also showed tdTomato activity at 4 weeks. At later time points (8 weeks and 1 year), tdTomato activity persisted in the OB and became more extensively expressed across the entire piriform cortex (Figure 1a). At P4 and P10, coronal sections of the OB showed tdTomato expression in dispersed cells in the glomerular layer and the granular layer (GrO) (Supplementary Figure S1a). At 1 year, sagittal sections showed tdTomato activity was more extensively distributed throughout the main olfactory bulb (MOB), with the GrO and external plexiform layer (EPL) exhibiting the strongest signals. The accessory olfactory bulb (AOB) also showed substantial tdTomato activity. Strong tdTomato signal was also seen in the surrounding olfactory-related regions including the dorsal tenia tecta (DTT) and the medial part of the anterior olfactory area (AOM) (Figure 1b). Additionally, at 1 year, tdTomato signal was observed in broader brain regions including the anterior commissure (ac), fornix (f), endopiriform nucleus (En), nucleus accumbens (NAc), olfactory tubercle (Tu), and the molecular and granular cell layers of the dentate gyrus (MoDG and GrDG) (Figure 1c,d).

To verify that the observed *Col10a1*-Cre activity in the brain was not due to random genomic integration, the *Rosa26-tdTomato* reporter line was combined with the inducible *Col10a1^Cre-ERT2^* line to generate *Col10a1^Cre-ERT2/+^*; *R26^td/+^* mice. Tamoxifen (TX) was injected at P4, P6, and P8 to induce Cre activity. At P9, tdTomato was detectable in OB cells of *Col10a1^Cre-ERT2/+^*; *R26^td/+^*; *Ctnnb1* cKO mice (Supplementary Figure S1b). The presence of tdTomato activation in the two independent *Col10a1*-Cre strains (*Col10a1^Cre^* and *Col10a1^Cre-ERT2^*) confirmed the specificity of the *Col10a1*-Cre activity. Interestingly, *Ctnnb1* was also found to be widely expressed in the OB and hippocampus [32]. The co-expression of the *Col10a1* and *Ctnnb1* genes in the OB and the DG of hippocampus provided the possibility of genetically altering the expression of the *Ctnnb1* gene by using *Col10a1*-driven genetic tools, specifically in these two regions in the adult stage but not in the embryonic stage.

### 2.2. Col10a1-Cre Mediates Inactivation of Ctnnb1 in the OB and Hippocampus

Given the specific expression of *Col10a1*-Cre in olfactory regions and the hippocampus, we generated *Col10a1^Cre/+^*; *R26^td/+^*; *Ctnnb1^−/−^* compound mutants to conditionally ablate *Ctnnb1*. The sequencing result of DNA extracted from the OB of one-year-old *Col10a1^Cre/+^*; *R26^td/+^*; *Ctnnb1^−/−^* mice (*Ctnnb1*^cKO^ mice) showed that the genome of some OB cells had only one LoxP site in the sequence between F1 and B2 primers with two primers flanking the *Ctnnb1-flox* structure [2]. This result indicates that *Col10a1*-Cre excised exon 3-6 of the *Ctnnb1* gene at or before one year old (Figure 1e).

### 2.3. Ctnnb1^cKO^ Mice Have Intact Food Localization Ability

**Figure 2:**
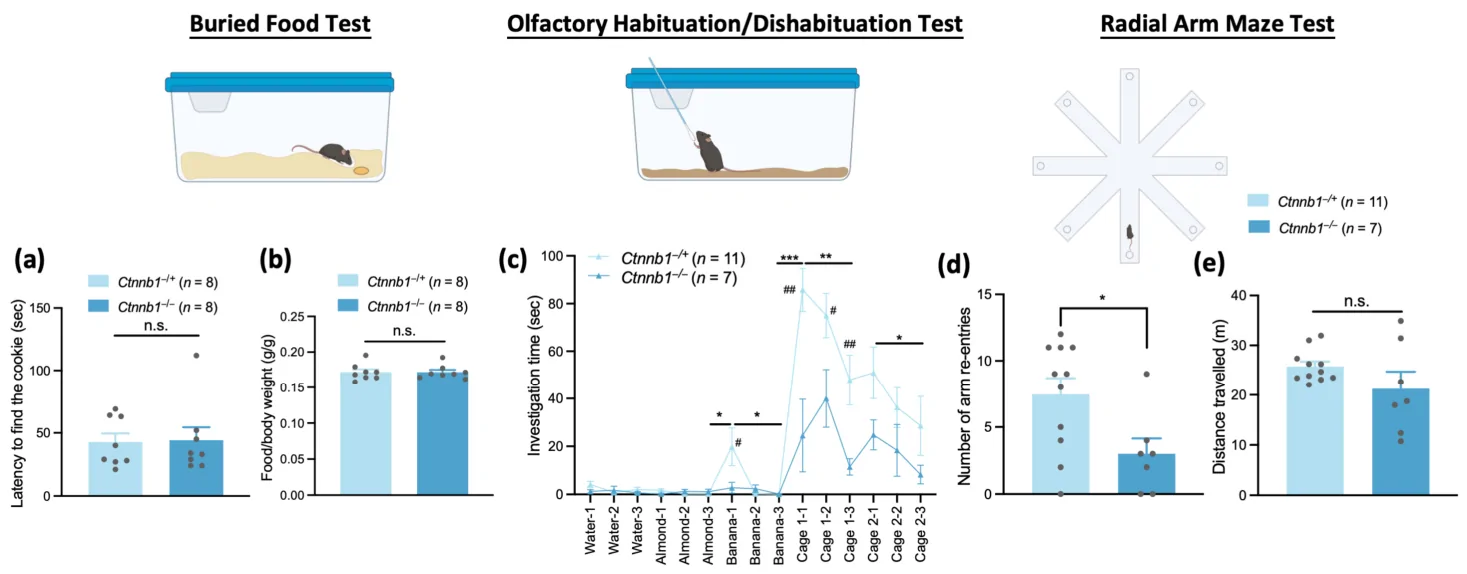
(**a**,**b**) *Ctnnb1*^cKO^ mice and heterozygous controls showed no difference in their Buried Food Test performance and basal food consumption levels. (**a**) Latency to excavate the buried cookie in the Buried Food Test in *Ctnnb1*^cKO^ mice and heterozygous controls. (**b**) Daily food consumption normalized by body weight in *Ctnnb1*^cKO^ mice and heterozygous controls. (**c**) *Ctnnb1*^cKO^ mice displayed impairments in odor discrimination and habituation in the olfactory habituation/dishabituation test. Time spent by the two strains of mice investigating the presented odors is shown. (**d**,**e**) *Ctnnb1*^cKO^ mice showed slight improvements in working memory in the Radial Arm Maze test on day 3. (**d**) Number of repeated entries into the arms before all food pellets were consumed. (**e**) Total distanced traveled by the two strains of mice during the 10 min test. Animal numbers (heterozygous control, *Ctnnb1*^cKO^): (**a**,**b**), *n* = 8, 8; (**c**–**e**), *n* = 11, 7. Values are mean ± SEM. *, **, and ***, *p* < 0.050, 0.010, and 0.001 in paired samples *t*-tests. #, and ##, *p* < 0.050 and 0.010 in independent samples *t*-tests. n.s., statistically non-significant in independent samples *t*-tests or Welch’s unpaired *t*-test.

Given the key roles of the olfactory bulb and hippocampus in olfaction and related behaviors, we used the buried food test (BFT) to assess whether *Ctnnb1*^cKO^ and heterozygous control mice could detect the volatile odor of familiar appetitive food and locate it beneath the bedding after food deprivation. We observed that the *Ctnnb1*^cKO^ mice took a comparable amount of time to locate and excavate the buried cookie compared to controls (Figure 2a, independent samples *t*-test, *p* = 0.905). Moreover, food consumption normalized to body weight did not differ between heterozygous control and *Ctnnb1*^cKO^ mice (Figure 2b, independent samples *t*-test, *p* = 0.984).

### 2.4. Ctnnb1^cKO^ Mice Display Odor Habituation and Dishabituation Impairments

The olfactory habituation/dishabituation test [36] was used to examine the ability of *Ctnnb1*^cKO^ and heterozygous control mice to detect and distinguish two neutral odors (almond and banana) and two unfamiliar social odors (from two different cages previously housing unfamiliar male mice) (Figure 2c). We observed no sustained investigation of sterile Q-tips during the 30 min habituation period or the initial water-control trials, suggesting that the increased investigation of subsequent odor stimuli was unlikely to reflect nonspecific interest in the Q-tip itself. A clear olfactory dishabituation effect was observed in heterozygous control mice towards to the first exposure to neutral odor (banana) and the first exposure to non-neutral odor (cage 1 odor), but not the almond or cage 2 odors (paired *t*-test; banana *p* = 0.032, almond *p* = 0.754, cage 1 *p* < 0.001, cage 2 *p* = 0.831). The olfactory disinhibition effect in *Ctnnb1*^cKO^ mice was diminished for neutral odors (paired *t*-test, almond *p* = 0.423 banana *p* = 0.240). This mouse strain still displayed mildly increased investigative behaviors on first presentation of the two social odors, but the changes were not statistically significant (paired *t*-test, cage 1 *p* = 0.156, cage 2 *p* = 0.096).

To examine if the two mouse strains can habituate upon repeated exposure to the same odor, the investigation time during the second and third presentations was compared to the investigation time of the first presentation for each odor. For the heterozygous control mice, the investigation times during the second exposure to the banana odor (paired *t*-test, *p* = 0.031) and during the third exposure to banana, cage 1, and cage 2 odors were significantly shorter than during the first odor exposures (paired *t*-test, banana, *p* = 0.030, cage 1, *p* = 0.006, cage 2, *p* = 0.037), demonstrating an olfactory habituation effect. In contrast, *Ctnnb1*^cKO^ mice did not display any significant decrease in investigation time during the second or third presentation of any odors (paired *t*-test, *p* > 0.050), indicating impaired odor habituation.

Notably, we observed that the response to novel odors in *Ctnnb1*^cKO^ mice was significantly weaker than in the heterozygous controls. The *Ctnnb1*^cKO^ mice showed significantly less investigation time during the first trial of banana odor exposure, all three trials of cage 1 odor exposure, and the first trial of cage 2 odor exposure when compared to heterozygous controls (independent samples *t*-test, banana-1 *p* = 0.047, cage 1-1 *p* = 0.002, cage 1-2 *p* = 0.035, cage 1-3 *p* = 0.006, cage 2-1 *p* = 0.054).

### 2.5. Ctnnb1^cKO^ Mice Display Modestly Enhanced Working Memory in the Radial Arm Maze Test

A modified Radial Arm Maze (RAM) test [37,38] was used to test the working memory of *Ctnnb1*^cKO^ and heterozygous control mice, in which the number of arm re-entries until all food pellets in all eight arms were consumed was recorded. *Ctnnb1*^cKO^ mice exhibited significantly less arm re-entries compared to the heterozygous controls on day 3 of testing (Figure 2d, independent samples *t*-test, *p* = 0.048), indicating a more efficient search strategy and thus better working memory. Importantly, the two mouse strains traveled similar distances during the RAM test (Figure 2e, Welch’s unpaired *t*-test, *t*_6_._98_ = 1.255, *p* = 0.250), suggesting the decreased arm re-entries was not due to decreased overall locomotor activity or exploratory drive. This result demonstrates a surprising, modest improvement in working memory in a commonly used behavioral task.

## 3. Discussion

In this study, we report *Col10a1*-Cre-driven conditional β-catenin KO in selective brain regions covering mostly olfactory processing areas in adult mice. We showed that β-catenin KO in these regions in adult mice impaired odor discrimination and habituation, but modestly improved working memory in the RAM test. The sense of smell is the dominant sensory modality in rodents, and they rely heavily on olfaction to recognize food sources and navigate their living environment [39]. Therefore, it was surprising to find that the β-catenin KO induced olfactory impairments in mice which were accompanied by memory improvement in a food foraging task.

We firstly tested whether β-catenin KO affects odor discrimination. We found that 3-month-old *Ctnnb1*^cKO^ mice had impairments in their ability to detect novel odors and habituate to odors upon repetitive exposure. This finding is not unexpected, as we observed extensive *Col10a1*-Cre activity in olfactory-related brain regions including the OB and hippocampus. Our results suggest that *Ctnnb1* in these brain regions plays an important role in odor detection and habituation in adulthood. However, the exact cellular and molecular mechanisms by which β-catenin regulates odor detection and habituation in adulthood remains unclear. Here, we propose two possible ways β-catenin KO in these regions in adult brain could affect olfaction. First, Wnt/β-catenin signaling in the OB regulates synaptic formation between the axons from newborn ORNs and existing glomeruli in the OB. During OB embryonic development, Wnt/β-catenin signaling plays an essential role in the formation of proper synaptic connections between the OB and incoming axons from the ORNs [30]. In adulthood, the axons of newborn ORNs are constantly integrated to existing glomeruli in the OB, providing functional inputs to maintain and refine existing glomerular circuitry [40,41]. Disruptions in Wnt/β-catenin signaling may lead to incorrect innervation of glomeruli, leading to degraded precision in existing odorant mapping in the glomeruli circuit. Second, Wnt/β-catenin signaling in the OB may regulate the migration, differentiation, and circuit integration of neuroblasts generated in the SVZ. Blocking Wnt/β-catenin signaling via intraventricular injections of Dickkopf-1 in mice was shown to inhibit neurogenesis in the SVZ, leading to impaired olfactory memory [42]. Neuroblasts migrating from the SVZ to the OB via the rostral migratory stream (RMS) are ensheathed by astrocytic processes that express β-catenin and other membrane-cytoskeletal scaffolding proteins [43]. Astrocytes in the RMS are known to provide structural and metabolic support for the survival and migration of neural precursors [44]. After reaching the OB, neural precursors migrate radially and differentiate into periglomerular cells and granule cells, which are populations of inhibitory interneurons that are known to modulate the activity of OB principal projection neurons for stimuli discrimination and contrast enhancement [45,46,47]. In *Ctnnb1*^cKO^ mice, extensive *Col10a1*-Cre activity was seen surrounding the RMS area (Figure 1b). It is therefore likely that β-catenin KO in this region affects the migration of neural precursors into the OB, subsequently disrupting the OB inhibitory interneuron network and leading to impaired odor discrimination and habituation.

We recognize that the olfactory habituation/dishabituation test is a non-associative behavioral assay that relies on voluntary investigation of the odor source and therefore does not provide a direct measure of olfactory sensitivity or detection threshold. Reduced investigation may reflect changes in motivation, attention, exploratory behavior, or odor processing in addition to altered olfactory function. Future studies incorporating concentration-dependent odor detection threshold assays would provide a more sensitive assessment of olfactory sensitivity and help distinguish these possibilities. We also note that heterozygous control mice did not show clear dishabituation to the first presentation of almond or the second unfamiliar social odor (Cage 2). Because wild-type background-strain mice were not included as an additional control group, we cannot determine whether these responses reflect normal variability in the assay, stimulus-specific effects, or altered olfactory behavior associated with the heterozygous genotype. Therefore, the heterozygous controls should not be assumed to exhibit normal responses to all odor stimuli, and comparison with wild-type mice would be informative in future studies.

As rodents rely heavily on their sense of smell to locate food, we suspected that the *Ctnnb1*^cKO^ mice would show impaired food foraging efficiency because their ability to detect odors is compromised. To test this hypothesis, the two mouse strains were subjected to two behavior tests, BFT and RAM. Unexpectedly, we found that the *Ctnnb1*^cKO^ mice performed comparatively to the controls in BFT, despite showing impaired ability to detect and habituate to novel odors in the olfactory habituation/dishabituation test. It is important to note that the two olfactory assays conducted in this study probe different aspects of olfaction-guided behavior: the BFT assesses detection and localization of a familiar food odor after fasting, whereas olfactory habituation/dishabituation test assess responses to unfamiliar odors, including detection, discrimination, and habituation. Successful food localization therefore does not establish a normal olfactory detection threshold, while reduced odor investigation may reflect altered sensitivity, odor processing, motivation, or attention. Together, these findings support that the *Ctnnb1*^cKO^ mice have impaired odor-investigation behaviors under the tested conditions without a general inability to detect odors.

As we observed extensive *Col10a1*-Cre activity in the DG of *Ctnnb1*^cKO^ mice, we expected their spatial navigation and memory abilities would also be affected. We found that *Ctnnb1*^cKO^ mice displayed slightly better working memory than controls in the RAM test on day 3, which was unexpected as Wnt signaling is a crucial regulator of adult neurogenesis in the hippocampus [7], and is required for maintaining spatial navigation ability throughout the animal’s lifetime [48,49]. Therefore, it is surprising to find that the *Ctnnb1*^cKO^ mice exhibited better working memory in the RAM test. Some possible explanations for these observations are proposed. First, the observation that *Ctnnb1*^cKO^ mice were able to complete the BFT and RAM test suggests that the genetic manipulation adopted in this study might not have fully blocked the animals’ ability to smell and perform olfaction-related tasks. We found *Col10a1*-Cre activity was only sparsely observed in the OB at P4 and P10 (Supplementary Figure S1) but was more extensively seen in the OB and other olfactory-related regions at 1 year. This suggests that *Col10a1*-Cre activity is mostly postnatal and likely gradually reaches its highest levels in adult animals. Therefore, adult animals possibly still have intact functional OB and hippocampal circuits that developed prenatally and during adolescence, which are sufficient to support basic food foraging and navigation tasks. In addition, as *Col10a1*-Cre-driven *Ctnnb1* cKO in the postnatal brain is gradual, it is possible that other signaling pathways can functionally compensate for the loss of Wnt/β-catenin signaling. Moreover, the *Ctnnb1* cKO-induced olfaction impairments may have led to neuronal and behavioral adaptions that allow the animals to rely more on their memory rather than on primary olfactory inputs to locate food. The increased reliance on memory to perform the task may have strengthened their memory circuits and compensated for the possible adverse effects of *Ctnnb1* cKO, such as reduced neurogenesis. However, the improvement in working memory observed here was modest, and the RAM alone does not provide a comprehensive assessment of working-memory function. Future studies should incorporate complementary paradigms, such as delayed alternation in the T-maze [50], in which working-memory demand can be systematically varied by altering the retention delay, and delayed non-matching-to-sample tasks [51], which assess short-term retention and discrimination of odor information. Together, these assays would provide a broader assessment of the effects of *Ctnnb1* cKO across spatial and olfactory working-memory domains.

We recognize that the present assays do not determine whether altered olfaction-guided behaviors arise from altered sensory function in the peripheral olfactory epithelium, central brain processing, or both. We examined the Bgee database (https://www.bgee.org/, accessed on 30 September 2026) and found no reported *Col10a1* expression in the olfactory epithelium of C57BL/6 mice [52]. Because our *Ctnnb1* cKO mice were generated using the *Col10a1^Cre/+^*; *R26^td/+^* line, this suggests that recombination in the olfactory epithelium is unlikely and that the observed behavioral effects are more likely to arise from central *Ctnnb1* deletion in the OB–hippocampal circuitry. However, we did not directly assess *Col10a1* expression or *Ctnnb1* recombination in the olfactory epithelium of our mice and therefore cannot exclude a peripheral contribution. We therefore do not attribute the observed effects specifically to OB-hippocampal circuitry. Resolving the peripheral and central contributions remains a question for future targeted studies.

In this study, we demonstrated that *Col10a1*-Cre-driven *Ctnnb1* knockout in olfactory-related brain regions including the OB and the DG of hippocampus of adult mice impaired odor habituation/dishabituation abilities, but slightly improved working memory in the RAM test. Although the observations in the present study remain correlational, our results suggest that β-catenin in these brain regions critically regulates odor discrimination and memory abilities in mice. Alterations in neuronal proliferation, migration, or circuit integration represent plausible mechanisms underlying the observed phenotypes in mice with *Col10a1*-Cre-driven *Ctnnb1* deletion, given the ongoing adult neurogenesis in the OB and DG and the established roles of Wnt/β-catenin signaling in neural development and adult neurogenesis. Additional physiological, imaging, and histological studies will be required to determine how *Ctnnb1* deletion contributes to these phenotypes. The present study demonstrates the viability of selectively manipulating gene expression in olfactory-related circuits in adult mouse brain using *Col10a1*-driven Cre. This approach will be useful for studying the functions of specific genes and pathways in these regions in adulthood.

## 4. Materials and Methods

### 4.1. Mouse Strains

*Ctnnb1*^fl/fl^ (gift from Yingzi Yang, Harvard University), Rosa26 tdTomato (*R26^td/+^*), *Col10a1^Cre/+^*, and *Col10a1^Cre-ERT2/+^* mouse lines bred in C57BL/6 background were used in the study [2,34,35,53]. All animals were group-housed in individually ventilated cages (39.1 cm × 19.9 cm × 16.0 cm; L × W × H) kept in a controlled environment at 25 °C under a 12 h/12 h light/dark cycle with food and water provided *ad libitum*. Up to six littermates were housed together in each cage. Animal care and experimental protocols were approved by the Committee on the Use of Live Animals in Teaching and Research (CULATR No. 5526-20) of the University of Hong Kong. All experimental procedures were conducted in accordance with the Animals (Control of Experiments) Ordinance (Cap. 340) regulations by the Hong Kong SAR government. All experimental methods followed recommendations in the ARRIVE guidelines. *Col10a1*-driven *Ctnnb1* conditional knockout was achieved by combining the *Ctnnb1*^fl/fl^ allele with the *Col10a1^Cre/+^;R26^td/+^* constitutive reporter line. Mice were sacrificed at P4, P5, P10, 2 weeks, 8 weeks, or 1 year by pentobarbital sodium injection (100 mg/kg, i.p.) and their brains were rapidly extracted for histological examination or DNA sequencing.

The conditional *Ctnnb1*^fl/fl^ allele was also combined with the inducible *Col10a1^Cre-ERT2/+^;R26^td/+^*line, and tamoxifen (TX) was injected at P4, P6, and P8. Tamoxifen dissolved in corn oil was administered by intraperitoneal injection at a dosage of 0.1 mg/g body weight. The mice were then sacrificed at P9 by pentobarbital sodium injection (100 mg/kg, i.p.) and their brains were rapidly extracted for histological examination.

### 4.2. Histological Analysis

Harvested brain samples were fixed in 4% PFA at 4 °C for 16–20 h, cryopreserved with 30% sucrose at 4 °C for 72 h, and then embedded in O.C.T (optimal cutting temperature) compound (Tissue-Tek^®^ O.C.T. Compound, Sakura Finetek, Torrance, CA, USA) at −20 °C. Sagittal sections (50 μm) were cut using a CryoStar NX50 Cryostat (Thermo Fisher Scientific, Waltham, MA, USA). The brain sections were counterstained by DAPI (1: 5000) for 10 min at room temperature, and then scanned by Vectra Polaris (PerkinElmer, Hopkinton, MA, USA) and analyzed by ImageJ (FIJI, Version 2.14.0).

### 4.3. DNA Sequencing

The OBs were dissected from 1-year-old *Col10a1^Cre/+^;R26^td/+^*;*Ctnnb1^−/−^* mice. DNA was extracted by TRIzol™ Reagent (Cat. No: 15596026, Invitrogen, Waltham, MA, USA) according to the manufacturer’s protocol. Target DNA fragments were amplified by PCR using primers described previously [2] (Figure 1e), isolated by agarose gel electrophoresis, and then purified using Monarch^®^ DNA Gel Extraction Kit (Cat. No: T1020, New England Biolabs, Ipswich, MA, USA). DNA sequencing service was provided by Tech Dragon Limited, HKSAR, China.

### 4.4. Behavior Tests

Three-month-old male *Col10a1^Cre/+^*; *R26^td/+^*; *Ctnnb1^−/−^* (*Ctnnb1*^cKO^) mice and *Col10a1^Cre/+^*; *R26^td/+^*; *Ctnnb1^−/+^* (heterozygous control) mice were subjected to behavioral testing to assess olfactory and memory performance. Behavioral experiments were conducted using two cohorts of male littermates generated in separate breeding rounds. For each cohort, all eligible male offspring from three breeding pairs were included to provide age- and litter-matched comparisons and to minimize variability associated with breeding batch and developmental stage. Because the numbers of heterozygous control and homozygous *Ctnnb1*^cKO^ male offspring varied between breeding rounds, group sizes differed between cohorts. The first cohort (*n* = 8 control, *n* = 8 *Ctnnb1*^cKO^) underwent the buried food test and food consumption/body-weight analysis. A separate cohort (*n* = 11 control, *n* = 7 *Ctnnb1*^cKO^) underwent the olfactory habituation/dishabituation test, followed by three days of rest, one day of RAM habituation, and three consecutive days of RAM testing. All test trials were recorded with an overhead camera (GoPro HERO11) and the videos were analyzed with ANY-Maze (Version 7.66).

### 4.5. Olfactory Habituation/Dishabituation Test

The Olfactory Habituation/Dishabituation Test was performed as previously described with slight modifications [36]. Clean cages (L: 39.1 cm × W: 19.9 cm × H: 16.0 cm) with open wire lids were used for testing. Odors were prepared freshly on the day of testing including two social odors and three neutral non-social odors: (1) distilled water, (2) almond extract (Rayner’s, UK, 1:100 dilution in distilled water), and (3) banana extract (Rayner’s, UK, 1:100 dilution in distilled water). For neutral odors, cotton Q-tips (20 cm) were dipped in the solution for 2 s before the trial. For social odors, cotton Q-tips were wiped onto different cages housing separate groups of unfamiliar wild-type male mice (unfamiliar cages 1 and 2). To avoid cross-contamination, the odors were prepared in a separate room and used Q-tips were immediately discarded in an enclosed bin after each trial. The wooden end of the Q-tip was inserted into the wire first to avoid contact between the tip and the cage lid. The Q-tips were held in place on the lid with binder clips. Test subjects were acclimatized to the testing setup for 30 min, during which a sterile cotton Q-tip with no odor was presented. During testing, mice were presented sequentially with the odors (distilled water, almond, banana, unfamiliar cage 1, unfamiliar cage 2) for three trials. Each trial lasted 2 min with an inter-trial interval of ~1 min. All test sessions were recorded by a video camera from a horizontal angle and later scored by a blinded experimenter. The accumulative time spent by the test subjects actively investigating the Q-tips (placing nose within a 2 cm radius of the Q-tip) during each trial was scored. Direct physical contact was permitted, and sniffing, licking, and occasional gnawing directed toward the Q-tip were all included in the investigation-time measure.

### 4.6. Buried Food Test

The BFT was performed as previously described with slight modification [36]. A clean cage (L: 42.5 cm × W: 26.6 cm × H: 18.5 cm) filled with a 3 cm layer of fresh beddings was used for the test. Mini-cookies (Hot-Kid Ball Cake from Want Want Group, ~1.5 g each) were scattered in the home cage of test mice for 3 consecutive days to ensure odor familiarization. The mice fasted for 24 h prior to the test. The mice were then allowed to acclimatize in the cage for 5 min and then temporarily transferred to a clean empty cage. One mini-cookie was buried about 1 cm beneath the surface in a random corner of the test cage. The mice were returned to the test cage and allowed 15 min to locate the hidden cookie. The latency to find the cookie was recorded. If the test mice failed to locate the cookie within 15 min, a latency of 900 s was recorded.

### 4.7. Radial Arm Maze Test

A modified RAM test was used [37,38] with eight arms (L: 35 cm × W: 8 cm × H: 15 cm) radiating from a central atrium (D: 24 cm). Mice were habituated to the testing apparatus for 1 day. Mice were then allowed to forage for food rewards in the maze for 3 consecutive days. All mice were food-deprived starting from 24 h before habituation and throughout the 3 days of testing to ensure they were motivated to explore the maze. During both habituation and testing, all eight arms were baited with a food pellet placed inside a dented cup (D: 3 cm) that was not visible from the central atrium. On each day, the mice were released into the central atrium and allowed 10 min to freely explore the maze and consume the food pellets. The number of arm re-entries until all food pellets in all eight arms were consumed was recorded. The total distance each mouse traveled in 10 min was calculated.

### 4.8. Statistical Analysis

All statistical analyses were performed using IBM SPSS Statistics (v29.0.2). Normality was assessed using the Shapiro–Wilk test. For between-group comparisons, homogeneity of variance was assessed using the Brown–Forsythe test. Independent-samples Student’s *t*-tests were used when variances were homogeneous; otherwise, Welch’s *t*-tests were applied. Paired-samples *t*-tests were used for within-group comparisons. A *p* value < 0.05 was considered statistically significant.

## Data Availability

The datasets used and analyzed during the current study are available from the corresponding author upon reasonable request.

## Supplementary Materials

The following supporting information can be downloaded at: https://www.mdpi.com/article/10.3390/ijms27208945/s1.
