# Supplementary material for: β-Catenin Knockout in the Olfactory Bulb and Hippocampus Reveals Dual Roles in Olfactory Discrimination and Working Memory
Source: Int J Mol Sci. 2026 Oct 9;27(20):8945. doi: 10.3390/ijms27208945 (PMC13704337; doi:10.3390/ijms27208945)
Supplement: Supplementary file 1 [file ijms-27-08945-s001.zip › ijms-4596466-supplementary.pdf]

Supplementary Figure S1

(a)

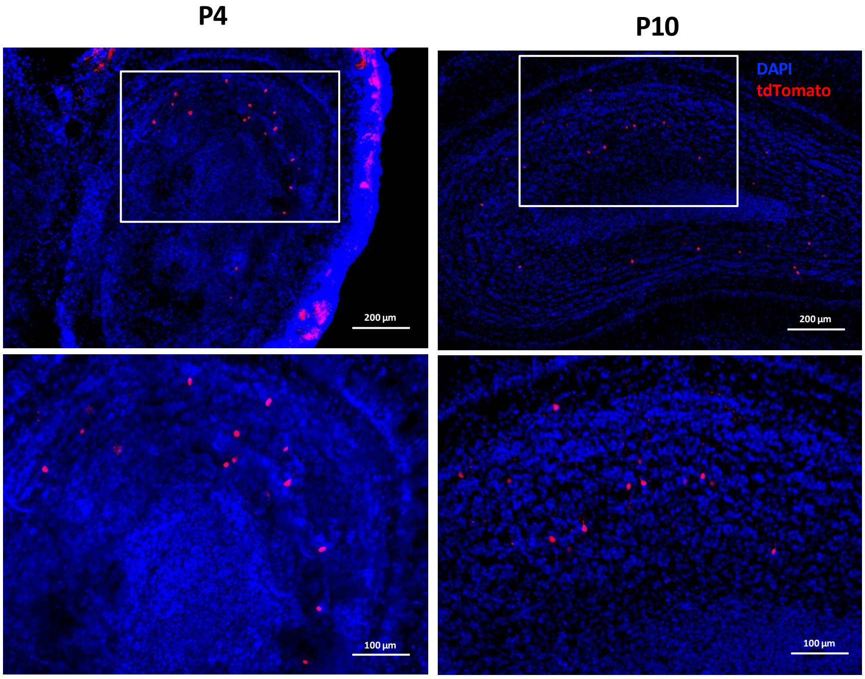

(b)

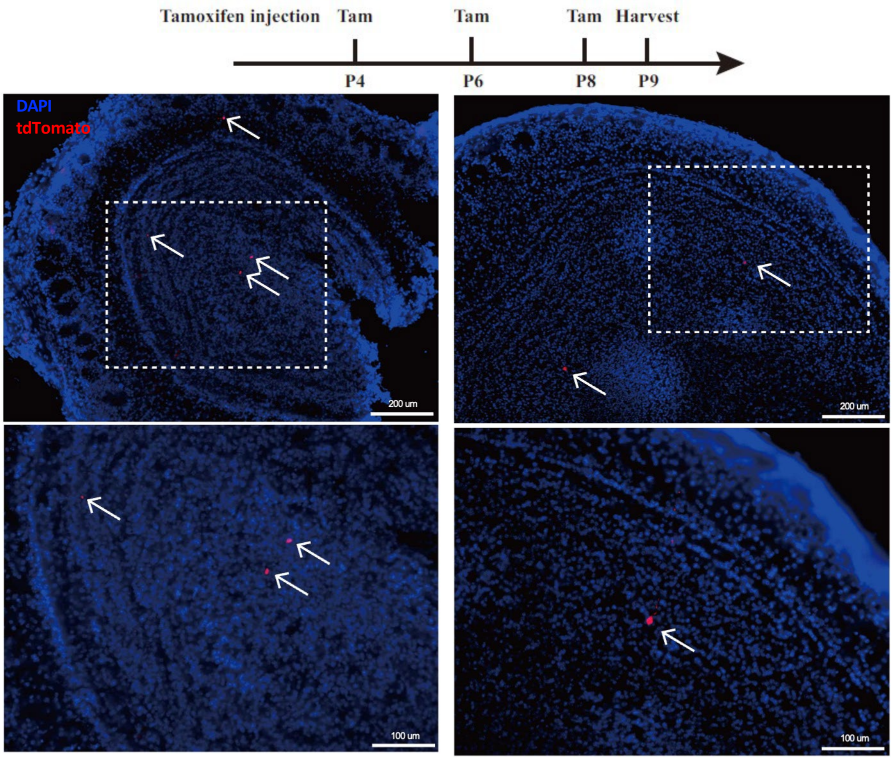

Supplementary Figure S1. tdTomato expression in olfactory bulb in *Col10a1*$^{Cre/+}$*;R26*$^{td/+}$ and *Col10a1*$^{Cre\text{-}ERT2/+}$*;R26*$^{td/+}$ mice.

(a) Expression of tdTomato (red) with DAPI (blue) in the olfactory bulb of *Col10a1*$^{Cre/+}$*;R26*$^{td/+}$ mice at postnatal day 4 and day 10.

(b) Expression of tdTomato (red) with DAPI (blue) in the olfactory bulb of *Col10a1*$^{Cre\text{-}ERT2/+}$*;R26*$^{td/+}$ mice at postnatal day 9 after tamoxifen injection at P4, P6 and P8. White arrows indicate representative tdTomato+ olfactory bulb cells.
